# Protein–Protein Interactions More Conserved within Species than across Species

**DOI:** 10.1371/journal.pcbi.0020079

**Published:** 2006-07-21

**Authors:** Sven Mika, Burkhard Rost

**Affiliations:** 1 Department of Biochemistry and Molecular Biophysics, Columbia University, New York, New York, United States of America; 2 Columbia University Center for Computational Biology and Bioinformatics, Irvine Cancer Center, New York, New York, United States of America; 3 Institute of Physical Biochemistry, University Witten/Herdecke, Witten, Germany; 4 NorthEast Structural Genomics Consortium, New York, New York, United States of America; Columbia University, United States of America

## Abstract

Experimental high-throughput studies of protein–protein interactions are beginning to provide enough data for comprehensive computational studies. Today, about ten large data sets, each with thousands of interacting pairs, coarsely sample the interactions in fly, human, worm, and yeast. Another about 55,000 pairs of interacting proteins have been identified by more careful, detailed biochemical experiments. Most interactions are experimentally observed in prokaryotes and simple eukaryotes; very few interactions are observed in higher eukaryotes such as mammals. It is commonly assumed that pathways in mammals can be inferred through homology to model organisms, e.g. the experimental observation that two yeast proteins interact is transferred to infer that the two corresponding proteins in human also interact. Two pairs for which the interaction is conserved are often described as interologs. The goal of this investigation was a large-scale comprehensive analysis of such inferences, i.e. of the evolutionary conservation of interologs. Here, we introduced a novel score for measuring the overlap between protein–protein interaction data sets. This measure appeared to reflect the overall quality of the data and was the basis for our two surprising results from our large-scale analysis. Firstly, homology-based inferences of physical protein–protein interactions appeared far less successful than expected. In fact, such inferences were accurate only for extremely high levels of sequence similarity. Secondly, and most surprisingly, the identification of interacting partners through sequence similarity was significantly more reliable for protein pairs within the same organism than for pairs between species. Our analysis underlined that the discrepancies between different datasets are large, even when using the same type of experiment on the same organism. This reality considerably constrains the power of homology-based transfer of interactions. In particular, the experimental probing of interactions in distant model organisms has to be undertaken with some caution. More comprehensive images of protein–protein networks will require the combination of many high-throughput methods, including *in silico* inferences and predictions. http://www.rostlab.org/results/2006/ppi_homology/

## Synopsis

The IntAct database contains about ten large-scale data sets of protein–protein interactions. Each set contains thousands of experimentally observed pair interactions. Most pairs were observed in yeast (Saccharomyces cerevisiae), fly (Drosophila melanogaster), and worm (Caenorhabditis elegans). These interactions are often perceived as model organisms in the sense that one can infer that two mouse proteins interact if one experimentally observes the two corresponding proteins in worm to interact. Here, the authors analyzed in detail how the sequence signals of physical protein–protein interactions are conserved. It is a common assumption that protein–protein interactions can easily be inferred through homology transfer from one model organism to another organism of interest. Here, the authors demonstrated that such homology transfers are only accurate at unexpectedly high levels of sequence identity. Even more surprisingly, homology transfers of protein–protein interactions are significantly more reliable for protein pairs from the same species than for two protein pairs from different organisms. The observation that interactions were much more conserved within than across species was valid for all levels of sequence similarity, i.e. for very similar as well as for more diverged interologs.

## Introduction

### Experiments Peek at Complete Protein–Protein Networks

The faster large-scale sequencing projects determine the alphabet of life, the higher the pressure to determine some of the actual processes that make life what it is. The understanding of functional relations among all proteins is essential to understanding how cells work. Recent breakthroughs in experimental high-throughput techniques have begun to peek at complete protein–protein interaction networks of entire organisms (Table S1). One central method is to use yeast two-hybrid (Y2H) assays [1] that are based on a genially simple idea: first, separate two domains (activation and DNA-binding) of a transcription factor that activates a reporter gene, then merge each of the two domains to a different protein (A and B) [2,3]. If A and B interact, the two transcription domains will merge, and thereby activate the reporter gene that will be detected. The difficulty of using Y2H is in mastering the details of the experimental setup. Other high-throughput methods to detect protein–protein interactions, such as phage-display assays [4], tandem affinity purifications (TAP) [5,6], co-immunoprecipitation, and affinity chromatography [2,7–9], are also commonly used. An important advantage of using Y2H over these other high-throughput techniques is the ability to measure physical interactions between proteins as opposed to pure functional associations. Also, Y2H experiments work with physiological conditions, i.e., conditions that resemble those in eukaryotic cells [2,3,10,11]. Ito et al. [12] and Uetz et al. [13] first scanned large fractions of the yeast proteome for protein–protein interactions. Others added further interactions: Ho et al. [14] used mass spectrometry and Gavin et al. [15] used TAP. Protein networks in the fly (Drosophelia melanogaster) have been targeted through three different Y2H studies [11,16,17], in the worm (Caenorhabditis elegans) through one [18], and a large subset of about 1,500 human protein network relations were detected through TAP [19]. These data bear deeper insights into cellular processes.

### Today's Data Are Incomplete and Not Fully Reliable

Y2H systems are not 100% accurate; they, for instance, identify many putative interactions that cannot be confirmed by other studies. One reason for false positives (interactions incorrectly postulated) is that the two proteins A and B may activate the reporter gene directly without having to interact [3]. The Margalit group has estimated the false positive rate in high-throughput Y2H assays to be about 50% [20]; the Eisenberg group has arrived at the same estimate through measuring the reliability of interactions in the Database of Interacting Proteins [21]. Y2H experiments also do not achieve complete coverage, i.e., they miss many interactions. Conversely, false negatives (missed interactions) might result from the particular experimental setup (which may prevent the interaction between A and B) or from problems in the assembly of the two transcriptional domains (activation and DNA-binding) needed for Y2H. These problems do not prevent Y2H from evolving as one of the major experimental probes for interactions; they do, however, imply that today's data sets are neither complete nor fully accurate [20,22]. One of the strong arguments in favor of large-scale Y2H experiments is that they are more systematic and much less driven by happenstance than hypothesis-driven, detailed experiments.

### Known Interactions Are Expanded through Homology-Based Inference

**Figure 1:**
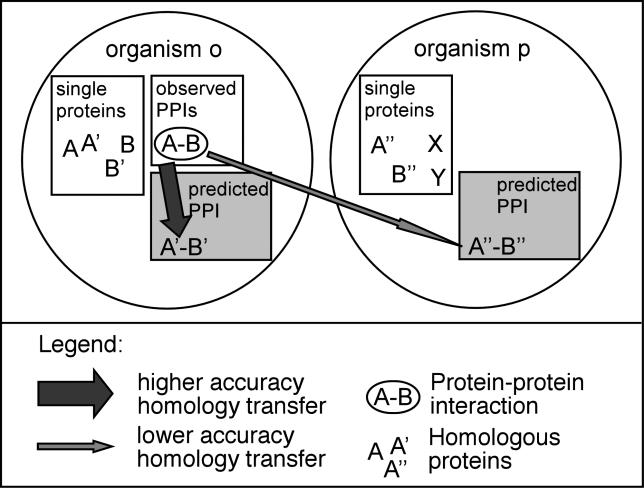
Concept of Homology Inference and Interologs Interologs are two pairs of protein interactions that fulfill the following conditions: (A interacts with B) + (A is similar to A′) + (B is similar to B′) → (A′ interacts with B′). All quadruples (A, B, A′, B′) for which this relation is true are referred to as interologs [37,79]. To illustrate our analysis, we have to extend this simple relation. Assume that a physical protein–protein interaction (PPI) between proteins A and B is observed in organism o. If A and B are both sequence similar (above a certain threshold) to two other proteins A′ and B′ in the same organism o, we should be able to infer the physical interaction between A′ and B′. Note that both pairs, A/A′ as well as B/B′, have to be above the particular similarity threshold for us to be able to make this inference. Thus, we neither use an average similarity of both pairs (A/A′ and B/B′) nor a minimum similarity for just one pair (A/A′ or B/B′). Now let us assume that we have another pair of proteins A″ and B″ in another organism p, and that both are as similar to A and B as are A′ and B′, respectively. One of our findings was that homology transfers A-B → A′-B′ were more reliable than those from A-B → A″-B″.

Evolutionary connections help explain the rapid success of molecular biology: we can study a particular protein in a simple bacterium and learn about the function of the same protein in multicellular eukaryotes. This idea enables us to use model organisms to predict protein structure [23–25], subcellular localization [26], enzymatic activity [27–29], and other aspects of protein function [30–34]. The same principle is frequently applied to the extension of interactions (Figure 1): Assume that two proteins A and B are experimentally observed to bind in organism o, and that alignment methods identify related protein pairs in organism o (A′-B′) and in organism p (A″-B″). Can we infer that the pairs A′-B′ and A″-B″ also interact with each other? The Vidal group [10] has investigated how yeast interactions detected by Ito [35] and Uetz [13] map to interactions in worm. They concluded that at BLAST E-values <10^−10^, only 16%–30% of the yeast interactions are transferable [36]; similar results were reported by the Gerstein group [37]. Although homology inference is common practice, no large-scale study has ever estimated levels of accuracy and coverage for physical interactions. A particular aspect of this question relates to paralogs and orthologs. Two proteins are often considered as paralogs when they originate from the same organism and differ in function. Paralogs are assumed to have arisen from gene duplication followed by the specialization and drifting away of one of the copies, while the other copy has maintained its original function. Orthologs, on the other hand, are described as two proteins with largely identical function and a common ancestor that reside in different organisms [37–39]. Applied to homology-based inference of interactions, a common assumption is that interactions are more conserved between orthologs than between paralogs [40–42], i.e., interactions are more conserved between than within organisms. If true, model organisms would be ideal for the study of interactions.

### Focus on Transient Physical Interactions (PPIs)

One important difference between Y2H and TAP is that while Y2H aims at the detection of physically interacting proteins, TAP identifies large groups of proteins that are associated, for instance, through a common pathway [43]. Most high-throughput techniques resemble TAP in the sense that they reveal association rather than physical interaction. To illustrate this difference, assume we hypothesized that co-expressed proteins interact physically, and we wanted to use this hypothesis to predict physical interactions directly from co-expression data. Assume further that six proteins are strung together in a linear pathway (1 binds 2, 2 binds 3, etc.), and that all six are co-expressed. Of the 15 [N*(N − 1)/2] possible interactions, only 5 (N − 1) are physical, i.e., only 33% of the co-expressed proteins interact. Since most pathways involve many more than six interactions this example is likely to significantly underestimate the actual problem. In other words, even if all physically interacting proteins were co-expressed, predictions of interactions based on such association alone would still be more often wrong than right. This significantly constrains the way in which we can use association-type data to analyze physical interactions. In order to emphasize our focus on physical interactions, we used the abbreviation PPI for transient physical protein–protein interactions (as opposed to functional associations as measured by TAP-like data, and as opposed to permanent physical interactions between, e.g., two different domains or two different chains of the same protein [44]).

### Coping with the Dilemma of Incomplete Data Sets

**Figure 2:**
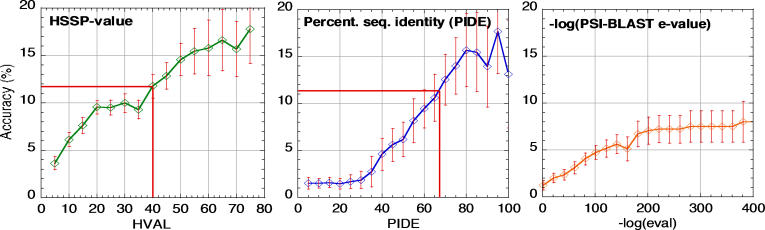
Sequence Conservation of PPIs The performance of homology transfer was evaluated with the data sets in Experiment 1 (Table 4). Each panel plots the conservation (accuracy of homology transfer) using a different measure for sequence similarity: HVAL (Equation 1), PIDE (percentage pairwise sequence identity), and the PSI-BLAST E-value. It is surprising that even at high similarity thresholds (PIDE > 50; HVAL > 30), accuracy remained low and never reached levels of 20%. This behavior was partially explained by our overlap analysis: for low overlap (Equations 2 and 3) between datasets, we expect a low accuracy. Numbers at HVAL = 40 (which equals a PIDE of 68 at an alignment length of 100 residues) were marked with red lines. HVAL = 40 is the point, where the overlap-values (Equation 3) for two identical datasets seem to indicate a zone of > 70% data consistency (see Table 3). Error bars for the three plots were calculated by bootstrapping over the PPIs in the source datasets (see Methods section).

How can we evaluate accuracy and coverage of homology transfer (Figure 1) of interactions if the data are incomplete? An extreme stance is to simply not assess the performance at all. The rationale is simple: assume a method inferred that A″ and B″ in Figure 1 interacted without any experimental evidence for this interaction. May be the inference was wrong; it also may just have been a new *in silico* discovery not yet identified by experiments. If the set of all interactions were complete, the absence of an observation would imply noninteraction. Although there is currently no such complete set, we challenge that the performance of homology transfer has to be estimated somehow to render a tool that is controllable in the context of genome annotation pipelines. Here, we took the opposite radical stance by treating all interactions that have not been observed as nonexisting. While this is obviously wrong, we assume that today's incompleteness is not systematic. If true, our results will simply underestimate the quantities that we measured, but will correctly capture relative values (such as that homology transfer is half as accurate at ~40% sequence identity as at ~60%, Figure 2). We also did not merge data sets that measure functional association (e.g., TAP) with those that measure physical interaction (e.g., Y2H). Instead, we regarded only physical interactions as positives.

**Figure 3:**
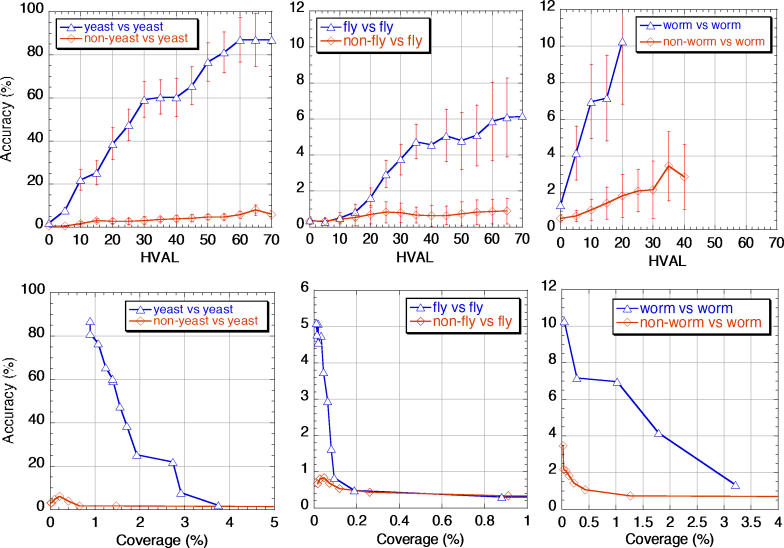
Performance of Homology Transfer Plots compiled for experiments 2–7 in Table 4. Each of the upper three graphs stands for one particular organism o and shows two plots: (1) Use all known PPIs (large-scale and small-scale) of organism o to find Y2H large-scale detected PPIs in the same organism (but from different experiment, blue line). (2) Use all PPIs (large-scale and small-scale) of all other organisms (not o) to find PPIs detected by Y2H in o (red line). Only organisms with available Y2H datasets in IntAct were chosen in order to be able to create complete interaction matrices for the target datasets (yeast, worm, and fruit fly). All error bars were calculated through bootstrapping over the source PPIs (100 times, Methods). Some lines end at certain thresholds because the counts for true positives and false positives were too low (< 30 true or false positives) to calculate accuracy (Equation 4, see Materials and Methods, often also referred to as specificity or precision). Figure S1 shows the correlation between the size of the error bars and the counts of true positives at each HSSP-value cutoff. The three bottom plots show ROC-like curves, where accuracy is plotted versus coverage for the exact same data as for the three upper plots. The figures demonstrate that for all levels of similarity, the accuracy of intraspecies predictions of PPIs is significantly higher than for predictions across two organisms.

**Figure 4:**
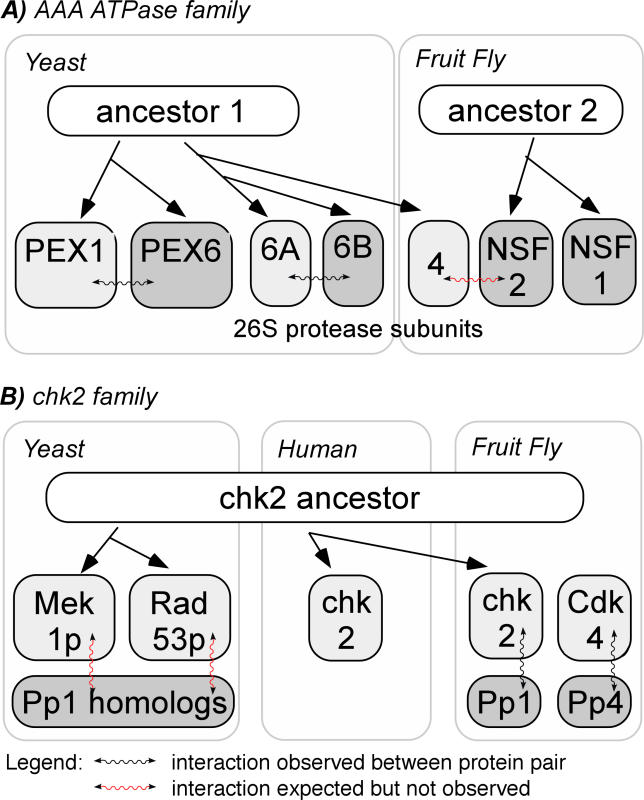
Interspecies Failure and Intraspecies Success of Homology Transfer (A) Same family, different ancestors, different PPI: Two yeast peroxisomal proteins (*PEX1* and *PEX2*) are closely related through their common ancestor protein and their function as AAA ATPases to the two yeast *26S protease regulatory subunits 6A* and *6B*. In the fruit fly, gene duplication of a second ancestor protein (the *NSF* ancestor) led to two distinct *NSF* proteins (*NSF1* and *2*). Since the ancestors for the NSFs (*NSF1* and *2*) and for the *26S protease subunits* were two different proteins, we conclude that despite their common biochemical function as ATPases, the different cellular functions of NSFs and 26S protease subunits also led to a distinct behavior with respect to protein–protein interactions. Therefore, neither *NSF1* nor *NSF2* were observed to bind to the *26S protease subunit 4*. (B) Same pathway, different functions, different binding: Evolutionary plasticity in the *chk2* family led to a diverse range of functions of these proteins while staying in the same pathway. For example *Rad53p* in yeast is a main player in the cell cycle checkpoint during mitosis, whereas *Mek1p* acts in the same position during meiosis. Also, *drosophila chk2* and human *chk2* act at different times during the cell cycle different from *Mek1p* and *Rad53p*. No *drosophila Pp1* homolog in yeast was found to interact with either *Mek1p* or *Rad53p*, even though *drosophila Pp1* was shown to bind to *drosophila* chk2.

Here, we presented the analysis of PPI in, to our knowledge, the largest data set investigated thus far. We defined and measured the overlap between different data sets, and analyzed the expected levels of accuracy and coverage for homology-based inference of PPIs depending on the level of sequence similarity. The most surprising finding originated from differentiating between intraspecies and interspecies inferences (o ≠ p in Figure 1), namely that PPIs are more conserved within than between organisms.

## Results/Discussion

### Different Experiments Overlap Very Little

If we want to homology infer PPIs between organisms, we first have to measure the overlap within organisms and then between organisms. We introduced such a measure (Equation 2 and Equation 3, see Materials and Methods) and applied it to assessing the overlap between datasets in IntAct [45]. A large overlap value implies high agreement between two experimental sets of interactions. Our definition of overlap takes into account that two data sets may not have used the same proteins thereby rendering a score that is, in principle, independent of the size of common subsets (see Materials and Methods section). The scores are straightforward when comparing different datasets within the same organism (Equation 2) because we only have to identify identical pairs of proteins. As noted before [22,46–49], the data sets overlap maximally for about 30% of all PPIs in yeast (*Saccharomyces Cerevisiae*) and much less for PPIs in fly (*Drosophila Melanogaster*, Table 1). Interspecies comparisons are trickier because we now have to identify the corresponding homologous pairs in the other organism. Equation 3 solves this problem by counting homologous instead of identical pairs of proteins; it is applicable to intraspecies and interspecies comparisons. A consequence of counting homologous rather than identical protein pairs is that the same data set no longer overlaps 100% with itself (Table 2), because the interaction between A and B may be detected while that between the homologs A′ and B′ may not be. The application of Equation 3 to the intraspecies comparison for yeast and fly datasets yielded similar results as the application of Equation 2 to the same datasets (Table 1). The overlap between different yeast datasets seems to be generally higher than that between different fly datasets. Finally, we merged datasets of different large-scale experiments for each organism and compared these pseudo-complete PPIs between organisms by using Equation 3 (Table 3). As expected the overlap between organisms was increased with increasing thresholds in what was considered homologous (Table 3; HSSP-value (HVAL)>40 highest, HVAL>0 lowest, Equation 1; note that the HSSP value (homology derived secondary structure of proteins) is an empirical measure for sequence similarity that empirically embeds the simple fact that high levels of sequence similarity are less meaningful for short than they are for long alignments). This increase in overlap was achieved by finding fewer matches (Table 3, empty cells). Conversely, the overlap was very low at levels of sequence similarity that mark the twilight zone of sequence-structure inference [25], i.e., the line above which most pairs of proteins have largely similar structure (HVAL>0, Table 3). In other words, overall fold similarity does not suffice to infer similarity in interactions.

**Table 1:**
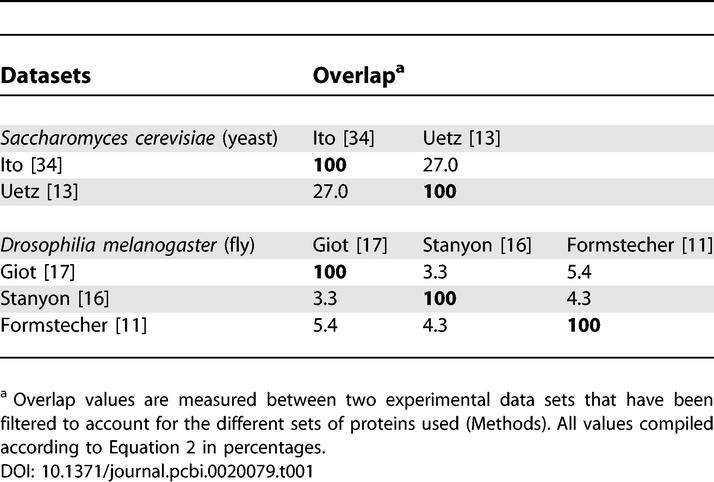
Identity-Based Overlap (Equation 2) between Original Experimental Y2H Datasets from Fly and Yeast

**Table 2:**
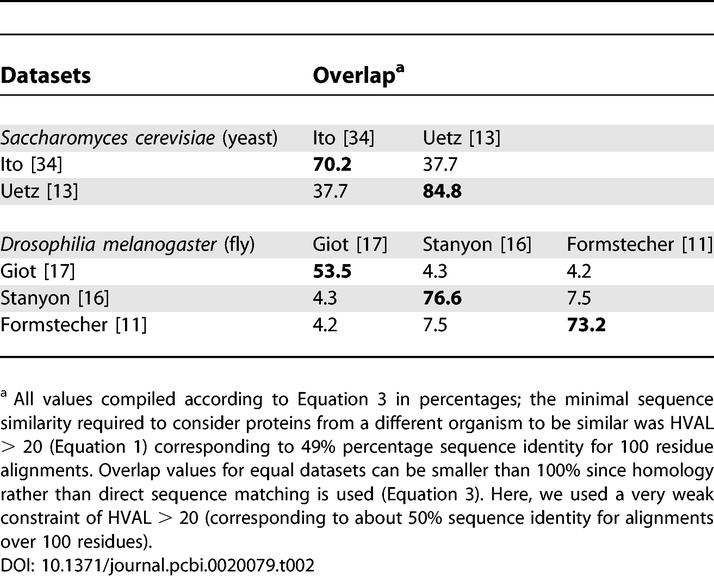
Homology-Based Overlap (Equation 3) between Original Experimental Y2H Datasets from Fly and Yeast

### Automatic Homology Transfer of PPIs Is Very Limited

We generated a homology performance plot (see Materials and Methods section) by comparing an unbiased, nonredundant data set (no two pairs of proteins in the set had significant sequence similarity (see Materials and Methods section) against the redundant set with all PPIs (note that we removed identical pairs even in this set, Table 4, Experiment 1). When using the observed PPI between two proteins (A-B), we applied the same sequence similarity threshold to identify both homologs (A/A′, B/B′) to infer the PPI between A′-B′. Pairs such as A-B′ or A′-B were not counted because those pairs could only be detected within the same organism and not across two species. Not surprisingly, the accuracy of homology transfer was proportional to sequence similarity (Figure 2). However, accuracy dropped rapidly already at very high levels of sequence similarity (e.g., at ~80% pairwise sequence identity, and below position-specific iterative basic local alignment search tool expectation values [PSI-BLAST E-values] < 10^−150^). Closer inspection of the HSSP formula (Equation 1) reveals that the curves for HSSP values and percentage sequence identity were very similar to each other. The problem with E-values largely originated from including short alignments, i.e., many of the proteins identified at very significant E-values (E < 10^−50^) might have been aligned to only small fractions of the source protein. This is a known limitation of E-values that cannot easily be normalized away because PPI interfaces may be rather short (i.e., even alignments of 20 residues in very long proteins may correctly reflect binding similarity). Although the small overlap between experimental data sets (Table 3) suggested that these estimates for accuracy at a given similarity threshold were most likely overpessimistic, the overlap scores also showed that at HVAL > 40, the consistency of the data was above 70% (Table 3). Therefore, our estimates at such high thresholds might be approximately correct; if so, the accuracy of homology transfer for high similarity (HVAL > 40, Percentage sequence IDEntity (PIDE) > 70) were just over 10% (Figure 2). Clearly, our findings suggested that automatic homology-based inferences of PPIs have to be taken with extreme caution.

### Homology Transfer Is Better within than between Organisms

Arguably [40–42], homology transfer is expected to be slightly better between organisms than within organisms. Instead, we observed the extreme opposite (Figure 3): at all levels of sequence similarity, and for all organisms with sufficient data, homology-inference was significantly more accurate for pairs of homologs from the same organism (intraspecies) than for pairs of homologs between different organisms (interspecies). In other words, if we experimentally observed the interaction between A and B in yeast, and if we found another pair of similar proteins A′ and B′ in yeast (not A-B′ or A′-B), as well as another pair A″ and B″ in fruit fly, then the interactions between A′ and B′ would be much more likely than those between A″ and B″. Consequently, yeast would be a rather poor model organism for the interaction network in fly.

Table 4 and Figures 2 and 3 clearly establish our main messages that intraspecies homology transfer is more accurate than interspecies transfer and that homology transfer is accurate only at unexpectedly high levels of sequence similarity. These results were stable with respect to different ways of processing the data for the experimental interactions. Changes that influenced the outcome insignificantly included the following alternatives.

### Results Were Stable with Respect to Details in Filtering Data

(1) Different sampling of intraspecies vs. interspecies: We allowed transfers of the type A-B to A′-B or A-B to A-B′ (see Materials and Methods section). The performance became significantly better for intraspecies PPI transfers, thus further widening the gap between intraspecies and interspecies transfers (Figure S2A). (2) Inclusion of transfers within the same data set: we included homology transfers within the same experimental dataset (see Materials and Methods section). The effect was very similar to those observed for different sampling (see #1), i.e., the gap was widened between intraspecies and interspecies inferences (Figure S2B). (3) We used TAP-like data (Table S1) as a constraint for the negatives. To illustrate this, assume that TAP pulled down a complex of six proteins. While we cannot infer that all 15 possible interactions are physical, all could be. Therefore, we ignored a false positive prediction (i.e., we did not count it) if we could find the interaction in those 15 TAP protein–protein pairs. The accuracy slightly increased for both yeast versus yeast (intraspecies) comparisons as well as for nonyeast versus yeast (interspecies) comparisons (Figure S2C). Note that yeast is the only organism with available TAP-like data. (4) We used a redundant dataset (instead of a nonredundant, bias-reduced set) from organism o (Figure 7) to hunt for interologs in organism p (Figure 7). The main message indicated by the results for this latter experiment stays the same as in our original procedure (see Materials and Methods section): Intraspecies comparisons are more accurate than interspecies comparisons. Because there were more samples in the dataset for organism o (Figure 7) and thus higher counts, the errors slightly decreased (Figure S2D).

### Examples

In the following, we presented a few representative examples that illustrate these points with more details than it is possible through averages over large data sets. Both show how homology transfer fails across species while it succeeds within an organism (Ao-Bo observed, A′o-B′o observed, A″m-B″m not observed).

#### Example 1: same family, different ancestors, different PPI.

The two peroxins *PEX1* and *PEX6* are known to functionally and physically interact in both human [50] and yeast [51–53] (Figure 4A). A particular mutation in human *PEX1* disrupts the interaction with *PEX6*, and appears directly linked to the Zellweger Syndrome, an autosomal, recessive peroxisome biogenesis disorder, in which the growth of the myelin sheath (the fatty cover of nerve cells in the brain) is strongly affected. Patients usually suffer from visual disturbances, high iron and copper blood levels, and enlarged livers [53]. Both proteins *PEX1* and *PEX6* belong to the ATPases associated with various cellular activities (AAA) family and are involved in the import of proteins into the peroxisome [52,53]. Thereby, the complex of *PEX1* and *PEX6* is associated with the cytoplasmic side of the peroxisomal membrane [51]. Searching for proteins that are sequence-similar to *PEX1* and *PEX6* within yeast at an HVAL > 20 (Equation 1, see Materials and Methods) brought up two *26S protease regulatory subunits, 6A* and *6B* (proteins A′o and B′o); experts have also classified both these yeast proteins as AAA ATPases (Figure 4A). The interaction between these two yeast proteins was surprisingly found in all Y2H large scale protein–protein interaction scans [13–15,35]. Using the same threshold (HVAL > 20) the closest proteins in fly were the *26S protease subunit 4* and the *NEM-sensitive fusion protein 2* (*NSF2*) (Figure 4A). The latter*—NSF2*— is a special form of the *NEM-sensitive fusion protein 1* (*NSF1*) and is fly-specific in the sense that it does not exist in yeast, worm, or human [54–56]. An interaction between *26S protease subunit 4* and *NSF2* was not found in any of our PPI *drosophila* datasets, nor has it been reported in the literature. *NSF2* is, among other things, responsible for exocytose through vesicle fusion by disassembling the postfusion SNARE protein complexes [54,57]. Like the other *PEX1* and *PEX6* relatives discussed so far, *NSF2* is also an ATPase [54]. A detailed phylogenetic analysis of all proteins in the AAA family has suggested three major subfamilies, one with NSF homologs (*NSF1* and *2*), one with the *26S protease subunits*, and a third with *p97/Cdc48p* homologs [56]. Most importantly these three subfamilies apparently did not arise from a common ancestor but rather, they evolved independently during speciation [56].

**Table 3:**
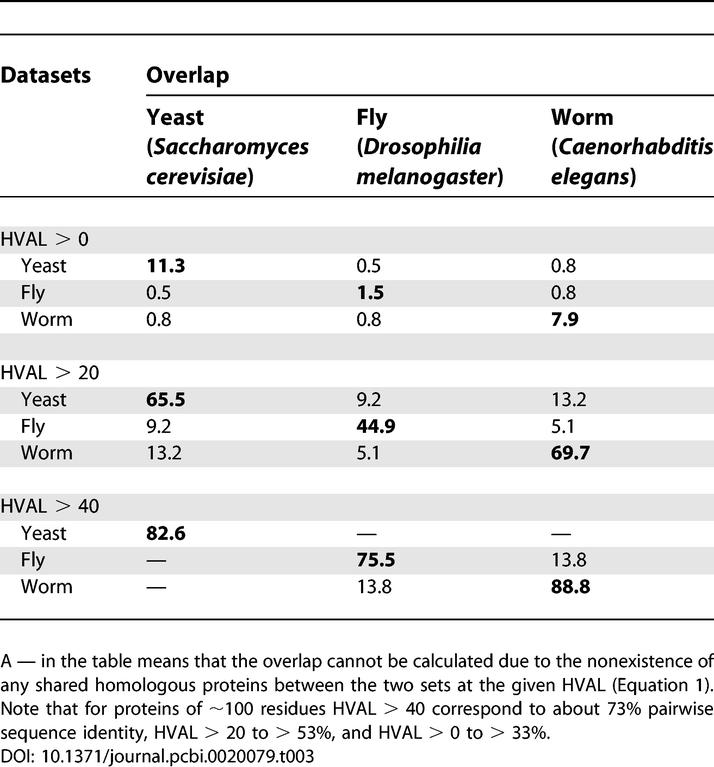
Homology-Based Overlap (Equation 3) between Merged Datasets for Different Similarity Thresholds

**Table 4:**
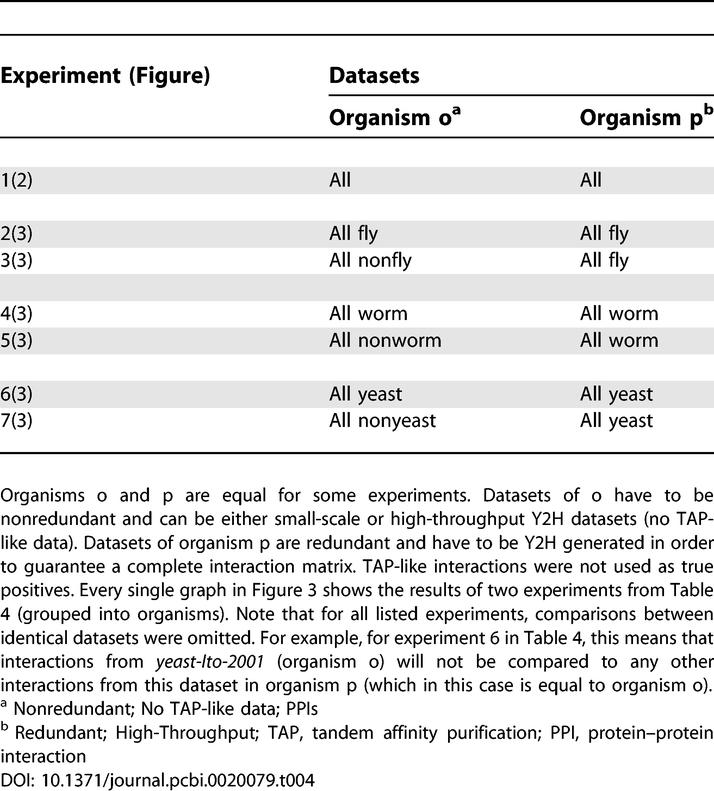
Datasets Used for Homology Performance Plots

This particular example illustrated how yeast may generally be a rather poor model organism for more complex species such as fly, worm or vertebrates. Proteins from these higher eukaryotes have to perform many different tasks in often highly specialized cell types (e.g., nerve cells). This might have lead to an evolutionary pressure to build new protein-interaction networks from the available protein building blocks (e.g., ATPase function). Thus, by only slightly altering the existing sequences, new binding properties were added to these proteins, while others were lost. A similar argument could be used to explain a likely poor homology transfer between fly and human or worm and human.

#### Example 2: same pathway, different functions, different binding properties.

The *drosophila Ser/Thr protein phosphatase 4* (*Pp4*) and the *cyclin dependent kinase 4* (*Cdk4*) were found in our small-scale dataset for *drosophila* PPIs. At HVAL>20, we found two sequence-similar proteins in fly, namely *Ser/Thr protein phosphatase alpha 2* (*Pp1*) similar to *Pp4*, and *chk2* similar to *Cdk4*; both these fly proteins (*Pp1* and *chk2*) have been shown to interact [16]. Fly *chk2* as well as its sequence relatives in yeast (*Mek1p* and *Rad53p*) and human are involved in cell-cycle checkpoints, which are signal transduction pathways that control the cell cycle and prevent the cell from further replication if the DNA double strand breaks, the DNA is incompletely replicated, or in case of other DNA damages [58–60]. A checkpoint can halt an ongoing mitosis or meiosis or even terminate it and induce apoptosis. A phylogenetic analysis of the *chk2* family members found that fly *chk2* and its yeast and human homologs stem from the same ancestor (Figure 4B). Nevertheless, it is also known that this family of proteins has a rather strong evolutionary plasticity in terms of the particular tasks of its members [60,61]. For example in yeast, *Mek1p* only controls the meiotic pachytene checkpoint by making sure that only homologous chromosomes recombine with each other [61], whereas yeast *Rad53p* controls mitotic cell replication and does not seem to be required for meiotic checkpoint control at all [60]. Also, the timing within the cell cycle is different for yeast *Rad53p* and its *drosophila* ortholog *chk2* [60]. This plasticity in the chk2 family might explain why many yeast proteins homologous to *drosophila Pp1* were not found to interact with either *Rad53p* or *Mek1p*.

### Sequence-Based Homology Transfer Is Limited Although Binding Sites Are Partially Conserved in Three-Dimensional (3-D) Structure

Recently, the Sali group analyzed the conservation of protein–protein binding sites on homologous and structurally aligned protein surfaces. They found that the differences in the localization of binding sites between homologous proteins are significantly smaller than the differences expected at random [62]. On the one hand, this result is similar to what we found for higher levels of similarity (Figure 3). On the other hand of very little similarity the difference between the 3-D–based results and ours lie most likely in the additional constraints implicitly used by the Sali group, namely that we know the 3-D structures and that we can focus in our alignment on all residues in the binding site. Using only sequence information, we cannot do this because binding residues close in 3-D may be separated considerably in sequence, thereby diluting the pattern of conservation picked up by alignment methods. However, for most PPIs from IntAct, we can neither label the binding site, nor do we have 3-D structural information. Therefore, we are limited to having to measure overall sequence similarity. If we were able to predict binding sites [63–66], we might improve homology transfer considerably.

## Conclusions

As demonstrated again by our overlap measure, today's datasets of PPIs are still rather inconsistent (Tables 1–3). The discrepancies were significantly smaller between yeast than between fly datasets (Tables 1 and 2). This finding also explains the much higher accuracy for intrayeast as opposed to intrafly or intraworm transfer. Why datasets of yeast appear more consistent than those of fly datasets remains speculation. One reason might be that measurements of protein–protein interactions are performed within yeast (Y2H) and are thus more precise for yeast proteins than for other species′ proteins, since those might behave differently in the unfamiliar yeast cell. Although incomplete and not fully consistent, PPI datasets are finally large enough to validate quantitative analyses. In particular, this enables a large-scale assessment of the performance of automated homology transfer for PPIs. Assuming that today's errors are largely nonsystematic, estimates for the performance of homology transfer will provide correct qualitative pictures, albeit the actual numbers will be overpessimistic. In the extreme regimen of comparing very similar pairs of proteins, we could establish that data sets appeared very consistent (Figure 2). Consequently, our estimates for the performance of homology transfer were likely to be relatively reliable in this regimen. Nevertheless, even for very high similarity, automated homology transfer was often mistaken; it approached random when approaching the sequence-structure twilight zone, i.e. the region in which sequence similarity no longer implies 3-D similarity (Figure 3). Although many interactions observed in one organism were not observed in another, similar interactions in the same organism (at similar levels of sequence similarity) were often observed (Figure 3). Consequently, our results challenge that using homology to transfer a protein–protein interaction from one organism to another is more difficult and less accurate than a transfer within the same species. This implies that distant model organisms have a limited value to unravel protein networks. We showed that these results are stable even when making major changes to the ways in which we analyzed the experimental data. Whether we used high- or low-confidence data, whether we allowed for same-set PPI transfers or not, whether we reduced bias or not, whether or not we filtered the negatives by TAP-like data about putative physical interactions, whether or not we restricted our analysis to limited inferences per family, we always observed the same: PPIs are more conserved within than across species. This discrepancy between intraspecies and interspecies conservation of interologs was valid for all levels of sequence similarity. Finally, we tested the ability of homology transfers to predict another functional annotation and then compared the performances of interspecies versus intraspecies comparisons thereof. We chose subcellular localization as an easily extractable and available protein feature. By using a list of proteins annotated for subcellular localizations from UniProt [67], we could show that there is no significant difference in performances for interspecies versus intraspecies homology transfers for this particular feature.

## Materials and Methods

### Data sets.

Several publicly available databases such as GRID [68], BIND [69], MINT [70], and DIP [71,72] gather information about interacting proteins in different organisms. For our analysis, we used the IntAct database [45], a protein–protein interaction resource maintained at the European Bioinformaics Institute (EBI) in Cambridge (http://www.ebi.ac.uk/intact/). IntAct uses the PSI format (extended markup language (XML)-tagged) to store data [73], fly [12–15], fly [11,16,17], worm [18] and human [19] as well as about 30 so called small-scale datasets, which are collections of results from many detailed experiments for different organisms. The largest small-scale dataset is that of human with about 38,000 interactions. Concerning the high-throughput datasets, IntAct carries detailed information about which proteins were used as baits and which proteins were used as preys, so that a complete interaction matrix can easily be reconstructed from these sets. Table S1 contains all protein–protein interaction datasets deposited in IntAct at the moment along with links to these datasets (small-scale and large-scale). The Giot [17], Ito [35], and Li [18] datasets contain some information about the level of confidence that was assigned to each interaction. For these three sets, we excluded everything from our analysis that either had a confidence-value of less than 0.4 (Giot: values range from 0 to 1) or those that were not in a so called “core” dataset of trusted interactions (Ito and Li divide their sets into core and full or core and noncore subsets, where core means a higher confidence in the measured interaction). Note that for the initial submission of this manuscript we had compiled all results for unfiltered data sets, i.e., we had included all experimental interactions; the results were qualitatively identical to those given here (data not shown).

### True positives and false negatives: focus on Physical Interactions = PPIs.

Technically, we realized our goal of exclusively focusing on PPIs through the particular way of labeling positives and negatives. We labeled as positives (true PPIs) only those pairs that were identified by experiments that target the detection of physical interactions (only Y2H experiments).

We then also assumed that these data for each organism was complete, i.e., we labeled all pairs as negatives that were not detected by Y2H.

### Measuring sequence similarity/homology.

The term homology usually implies an evolutionary relation in the sense of having a common ancestor. Strictly speaking, we cannot measure homology. Instead, alignment methods measure sequence similarity in some way or other. In our work the ranges of similarity were so high that the pairs of proteins were most likely homologous. We used BLAST and PSI-BLAST [74] to align all protein sequences in IntAct against each other (standard procedure [75]: 3 iterations at E<10-^10^ against filtered database of all proteins to build clean profiles, then one run with frozen profile against unfiltered database at E < 10^−3^, freeze profile again and run against all IntAct proteins). Then we extracted the PSI-BLAST E-values for each alignment, as well as the percentage of sequence identity (PIDE) and the distance to the HSSP curve, i.e. the HSSP-value [25,76,77] (HVAL). The HVAL is defined as:

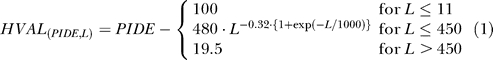
where L was the number of residues aligned between two proteins, and PIDE the percentage of pairwise identical residues. HSSP values consider both pairwise sequence identity and alignment length: the higher the value the more similar two proteins. Values around 0 typically imply that two proteins have similar 3-D structures and correspond to about 22% pairwise sequence identity at alignment lengths above 250 residues.

### Nonredundant data sets.

**Figure 5:**
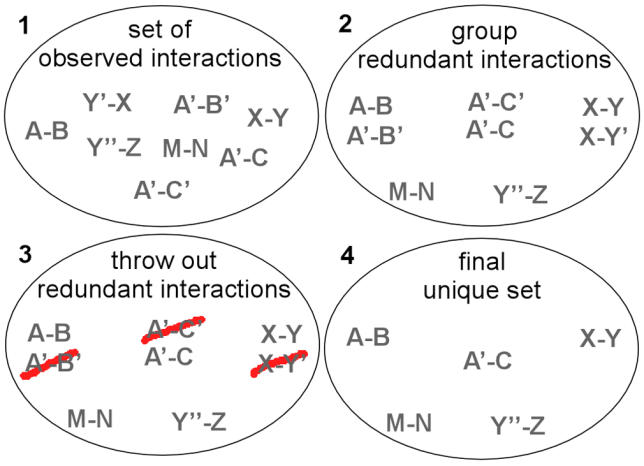
Creating Sequence-Unique PPI sets (1) Starting with a dataset of PPIs, we first cluster the data according to sequence similarity (apply a certain homology threshold) into sequence similar PPIs (2). Note here that the interactions A′-B′ and A′-C′ do not fall into the same cluster because B′ and C′ are unrelated. Thus, for two interactions (e.g., A-B and A′-B′) to be considered similar by our algorithm, both interacting proteins (A and B) have to be homologous to the two proteins of the other interaction (A has to be similar to A′ and B has to be similar to B′). (3) We randomly throw out all redundant interactions in each cluster so that only one PPI remains as a representative of each cluster. (4) Those representatives constitute the final unique dataset of PPIs.

We removed bias from PPI datasets by the following procedure (Figure 5). (1) Move down a list L of PPIs starting with pair A-B. (2) Group all interactions in this list into clusters of similar PPIs. Consider two distinct PPIs as similar only if both partners of the first interaction are homologs to the respective protein in the second interaction. For instance, let A′ be a homolog of A, and B′ be a homolog of B. Then all interactions A′-B, A′-B′, and A-B′ will fall into the same group as the interaction A-B. Note that this also means that any interaction A-C will not end up in this group if C is not a homolog of B. Here, we used a very conservative criterion for homolog, namely HVAL > 0 (Equation 1). This threshold is conservative in the sense that it will also remove nonredundant pairs, i.e., many proteins that are actually not homologs. (3) Reduce each group formed in step 2 to one single representative PPI. (4) Continue working with the final unique (nonredundant) dataset.

### Identity- and homology-based overlap between datasets.

**Figure 6:**
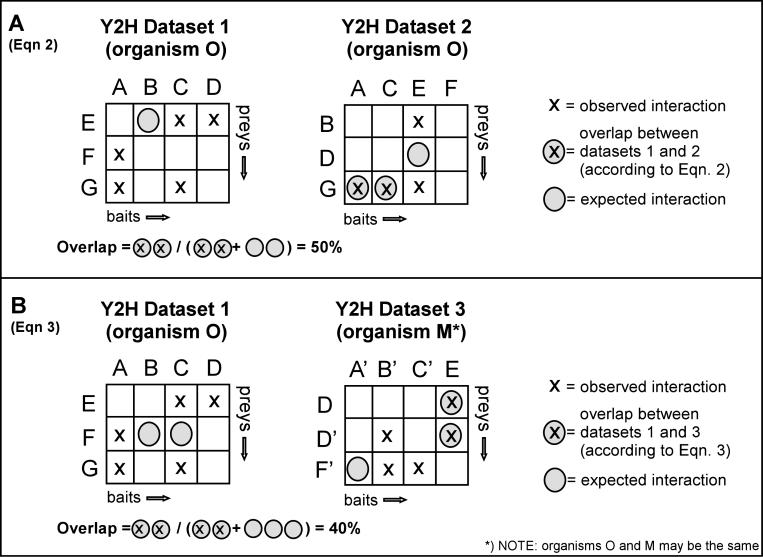
Ways of Calculating the Overlap between Two Y2H Datasets (A) Identity-based overlap between Datasets 1 and 2 according to Equation 2. Note that we can only calculate this score if both datasets are from the same organism. Starting with the observed interaction C-E in Dataset 1, we are trying to find the exact same interaction in Dataset 2. The following situations might occur: (a) C and E are also observed to interact in Dataset 2. (b) C and E are not observed to interact in Dataset 2. (c) It is impossible for C and E to be interacting in Dataset 2 due to either of these two reasons: (i) Either C or E are not part of Dataset 2 or (ii) C and E are either both used as preys or both used as baits in Dataset 2. Repeating the above procedure for all other observed interactions in Datasets 1 and 2, we finally calculate the identity-based overlap by dividing the number of common interactions found in Datasets 1 and 2 by the total number of expected interactions (observed and not-observed). (B) The same procedure as described above is applied to the two Datasets 1 and 3, which are now allowed to be from different organisms. The only difference to Equation 2 (A) is the usage of homology for comparing two PPIs instead of a binary decision scheme (PPIs identical or not-identical). Thus, starting with the interaction D-E from Dataset 1, we try to find possible homologous interactions (not only the identical PPI) in Dataset 3. The only two options in this example are D-E and D′-E (Dataset 3), which in our example are both observed in Dataset 3. Iterating through all observed interactions of Datasets 1 and 3 and summing up the expected interactions and the overlapping homologous interactions, we can then calculate the homology-based overlap (Equation 3). Note that any results from Equation 2 are not comparable to any results from Equation 3.

We defined two procedures resembling the Jaccard correlation to measure the overlap between two different datasets of PPIs in IntAct. Equation 2 defines the first measure; for clarity we refer to this measure as the identity-based overlap. This measure can only be applied to two PPI sets from the same organism.

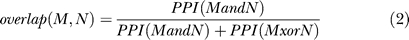
where *PPI*(*MandN*) is the number of PPIs that were detected in both sets (common PPIs) and *PPI*(*MxorN*) is the number of PPIs that were only detected in one of the two datasets (exclusive or). Figure 6A describes this procedure. Note that only those interactions contributed to the count of *PPI*(*MxorN*) that could possibly have been detected in both datasets. For example, if the PPI A-B is detected in dataset 1, but not in dataset 2, we only increase *PPI*(*MxorN*) by one, if A and B were both included in dataset 2. In other words, we completely ignored interactions A-B in one dataset, if either A, or B (or both) were not present in the other dataset. Given this definition (Equation 2), an overlap value of 0.5 means that every second PPI of dataset 1 is not present in dataset 2. Inversely, every second PPI from dataset 2 cannot be found in dataset 1. Furthermore, applying Equation 2 to calculate the overlap of one dataset with itself always results in 1 (100% overlap).

The second measure capturing an overlap between two interaction datasets was applicable to any two datasets, even if they were from different organisms. We referred to this measure as the homology-based overlap. It was defined as follows (Figure 6B):

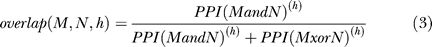
where *PPI*(*MandN*)^(*h*)^ is the number of homologous PPIs reported in both datasets considering a homology threshold of HVAL > h. Assume again that A is homolog of A′ and B of B′. If the interaction A-B is in dataset 1 and the interaction A′-B′ is in dataset 2, the count for *PPI*(*MandN*)^(*h*)^ will increase by one. The quantities *PPI*(*MandN*)^(*h*)^ and *PPI*(*MxorN*)^(*h*)^ are similar to those in Equation 2 with the simple caveat that we substituted identical pairs with homologous pairs, because there are no identical pairs between two different organisms. Unlike for Equation 2, when using Equation 3 to measure the overlap between a dataset and itself, the result usually happens to be < 1 (< 100%). For an explanation consider the following example. Assume that our dataset contains the interaction A(bait)-B(prey) along with another protein A′ (bait, homologous to A) that is not found to interact with B. The absence of A′-B will increase the count of *PPI*(*MxorN*)^(*h*)^ by one, thereby yielding a self overlap <1. On the one hand, for very high levels of similarity (say A and A′ have 99% pairwise sequence identity), the reduction from 1 can be interpreted as a reflection of the limitation of experimental accuracy. On the other hand, for low levels of similarity, the reduction is related to the fact that PPIs are simply not conserved between distant relatives. Note that we also investigated overlap when replacing HVAL (Equation 1) by PSI-BLAST E-values as a measure for sequence similarity. While the resulting numbers differed slightly, the trends that we reported remained the same (data not shown).

### Homology performance curves.

**Figure 7:**
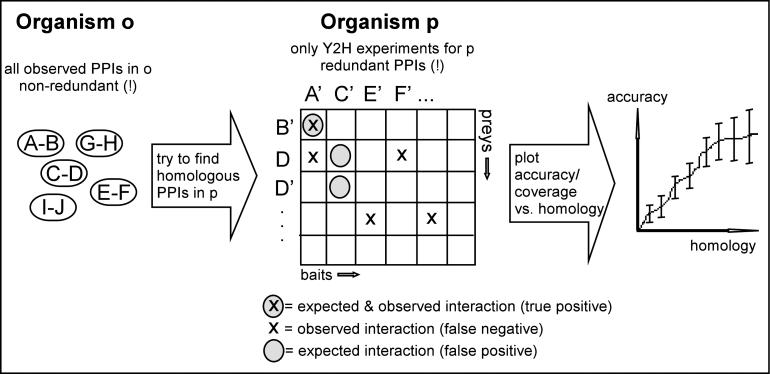
Evaluating Homology Inference of PPIs Starting with the entirety of observed interactions in any organism o (Y2H plus small scale experiments), we first reduce the sequence redundancy from this dataset as described in Figure 3. Then we try to find homologs in the organism p for each of the unique PPIs of organism o. Since we want to be able to conclude that every nondetected interaction in organism p does actually not exist in real life, we need to have a complete interaction matrix (baits × preys) for organism p. Thus, we are forced to exclude all small-scale data from the organism p dataset and remain with a merger of all (redundant) Y2H interactions for this organism. For each interaction A-B from organism o, we can face any of the following situations: (a) A homologous interaction A′-B′ can be found in organism p, (b) no homologous interaction can be found in p, or (c) It is impossible to detect an interaction of type A′-B′ in p because of one of the following two reasons: (i) either A′ or B′ are missing in the dataset for p or (ii) Both A′ and B′ are either preys or both are baits in the dataset for organism p. The latter case (c.ii) is illustrated by the interaction E-F in organism o, which cannot be detected in organism p only because E′ and F′ are both used as preys in the experiment. No counts for false positives are made for those cases. Adding the numbers of true positives (expected and observed PPIs), false positives (expected but not observed) and false negatives (observed interaction only in organism p) allows us to calculate accuracy and coverage for each homology threshold used to infer interactions (Equation 4). It is important to note that in the case where o = p, comparisons between two identical experimental PPI-sets are ignored (e.g. A-B in o′s set “*yeast-Ito-2001”* is not used to predict A′-B′ in p′s set “*yeast-Ito-2001”*; o = p = yeast).

For given levels of sequence similarity, we monitored and plotted the accuracy of inferring PPIs through homology from one dataset to another. The procedure is described in Figure 7.

The resulting curves can be interpreted as the degree to which PPIs are evolutionarily conserved. In a more technical sense, the curves reflect the performance of homology transfer of PPIs (Figure 1). The HVAL (Equation 1) determined the minimal similarity between A and A′, as well as between B and B′. Other ways of considering two pairs of interacting proteins as related, for instance the arithmetic or geometric average of both HVALs (A/A′ and B/B′), led to a slightly worse performance of our homology inferences, i.e. the curves were similar albeit lower overall (data not shown). Note that each large-scale Y2H data set (Table S1) should, by experimental design, contain a complete interaction matrix (preys × baits) that is, ideally, both fully correct and comprehensive for all the proteins tested in that experiment. Consider an interaction A-B from any dataset (small-scale or large-scale) of an organism o; if we find the homologs A′ and B′ in a large-scale dataset of another organism p, we can transfer the interaction property from A-B to A′-B′. In other words, by looking at the PPI between A and B (A-B), we simply predict that A′ and B′ also interact. Because of the complete interaction matrix that we are looking at for organism p, we can now also say whether this prediction was actually right or wrong. In particular, the prediction is correct, if we find the interaction A′-B′ in p and wrong if we do not find it in p plus A′ and B′ are on different axes of the interaction matrix (A′ = prey, B′ = bait or vice versa). In order to compare the performance of homology transfers across two organisms (o ≠ p) to the one for intraorganism transfers (o = p), we have to allow p and o to be the same. Therefore, in order to be able to compare results from both types of experiments (intraspecies versus interspecies), we have to apply the following restrictions to comparisons within the same species (o = p): Transfers from an interaction A-B to another PPI of the type A-B′ or A′-B (one protein identical, the other homologous) are not allowed since these cases are only observable in intraspecies predictions but not in interspecies transfers. Additionally for intraspecies predictions, we required that A-B and the predicted interaction (A′-B′) stem from different datasets (different Y2H experiments) in order to ignore possible homology-based assumptions about two PPIs within the same dataset. The problem here is that in case a research group found an interaction (e.g., A-B) through a Y2H scan, would they work harder to also find an interaction A′-B′ (A′ = homolog to A, B′ = homolog to B) or A′-B rather than an unrelated interaction (e.g., M-N).

### Accuracy and coverage.

We measured the accuracy (Acc) and coverage (Cov) for the inference (prediction) of interacting protein pairs by the standard formulas:

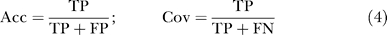
where TP are the true positives (i.e., physical interactions that are experimentally observed [e.g., by Y2H, note TAP-like relations are not included here] and that are also correctly inferred by homology). FP are the false positives (i.e., the pairs inferred through homology but not observed by Y2H experiments). Finally, FN are the false negatives (i.e., the physical interactions that have been observed but were not identified). We monitored levels of accuracy and coverage as a function of the sequence similarity between the proteins of known and those of unknown annotations. There is a trade-off between these two: the more restrictive the sequence similarity threshold, the more interactions will be inferred (higher coverage) at the expense of reduced accuracy; and the higher the threshold, the more will be right (high accuracy) at the expense of few inferences (low coverage).

### Error estimate.

The error in the estimates of accuracy and coverage were determined by bootstrapping [78] over the protein–protein interactions in the source datasets. In particular, we picked *n* interactions at random from the non-redundant source dataset and compiled the averages over a larger set with possibly many replicas of the same incidence. The levels of accuracy/coverage for different thresholds in sequence similarity were then calculated according to the procedure described above (Figure 7). For the bootstrapping, these two steps had been repeated 100 times before the standard deviation (sigma) for all levels of accuracy were calculated.

## Supporting Information

Table S1 Large-Scale Protein–Protein Interaction Datasets from IntAct (74 KB DOC)

Figure S1 Number of true positive counts versus HVAL Each curve shows the accuracy (red) as shown in Figure 3 and the number of true positives counted at a certain HSSP-value cutoff (green)
(72 KB DOC)

Figure S2 Results Are Stable with Respect to Variations in the Experimental Setup (A) Different sampling of intra- versus inter-species: we allowed transfers of the type A-B to A'-B or A-B to A-B' (see Materials and Methods section). The performance became significantly better for intra-species PPI-transfers, thus further widening the gap between intra- and inter-species transfers.
(B) Inclusion of transfers within the same data set: we included homology transfers within the same experimental dataset (see Materials and Methods section). The effect was very similar to those observed for different sampling (#1), i.e. widening the gap between intra- and inter-species inferences.
(C) Using TAP-like data (Table S1) as a constraint for the negatives. To illustrate this, assume that TAP pulled down a complex of six proteins. While we cannot infer that all 15 possible interactions are physical, all could be. Therefore, we ignored a false positive prediction (did not count it) if we could find the interaction in those 15 TAP protein-protein pairs. The accuracy slightly increased for both yeast versus yeast (intra-species) comparisons as well as for non-yeast versus yeast (inter-species) comparisons. Note that yeast is the only organism with available TAP-like data.
(D) We used a redundant dataset (instead of a non-redundant, bias-reduced set) from organism o (Figure 7) to hunt for interologs in organism p (Figure 7). The main message indicated by the results for this latter experiment (#4) stays the same as in our original procedure (see Materials and Methods section): intra species comparisons are more accurate than inter-species comparisons. Due to more samples in the dataset for organism o (Figure 7) and thus higher counts, the errors slightly decreased.
(153 KB DOC)

## Abbreviations

AAA: ATPases associated with various cellular activities
HVAL: measure for sequence similarity
PIDE: percentage sequence identity
PPI: physical protein–protein interaction
PSI-BLAST: position-specific iterative basic local alignment search tool
TAP: tandem affinity purification
Y2H: yeast two-hybrid
