# Supplementary material for: Protein–Protein Interactions More Conserved within Species than across Species
Source: PLoS Comput Biol. 2006 Jul 21;2(7):e79. doi: 10.1371/journal.pcbi.0020079 (PMC1513270; doi:10.1371/journal.pcbi.0020079)
Supplement: Supplementary file 1 [file pcbi.0020079.sg001.doc]

**Fig S1:** Each curve above shows the accuracy (red) as shown in Figure 3 (manuscript) and the number of true positives counted at a certain HSSP-value cutoff (green).
