# Supplementary material for: Protein–Protein Interactions More Conserved within Species than across Species
Source: PLoS Comput Biol. 2006 Jul 21;2(7):e79. doi: 10.1371/journal.pcbi.0020079 (PMC1513270; doi:10.1371/journal.pcbi.0020079)
Supplement: Supplementary file 2 [file pcbi.0020079.sg002.doc]

**Fig S2:** Results of varying the parameters of our experimental setup to measure the performance of homology inference of protein-protein interactions. Four experiments (A-D) were performed with differences to the experimental setup of Fig 3 (Methods section in manuscript) described below:

A: Different sampling of intra- vs. inter-species: we allowed transfers of the type A-B to A’-B or A-B to A-B’ (Methods). The performance became significantly better for intra-species PPI-transfers, thus further widening the gap between intra and inter-species transfers.

B: Inclusion of transfers within the same data set: we included homology transfers within the same experimental dataset (Methods). The effect was very similar to those observed for different sampling (#1), i.e. widening the gap between intra- and inter-species inferences.

C: Using TAP-like data (Table App_1, Supplementary Online Material) as a constraint for the negatives. To illustrate this assume that TAP pulled down a complex of six proteins. While we cannot infer that all 15 possible interactions are physical, all could be. Therefore, we ignored a false postive prediction (did not count it) if we could find the interaction in those 15 TAP protein-protein pairs. The accuracy slightly increased for both yeast vs yeast (intra-species) comparisons as well as for non-yeast vs yeast (inter-species) comparisons. Note that yeast is the only organism with available TAP-like data.

D: We used a redundant dataset (instead of a non-redundant, bias-reduced set) from organism o (Fig. 7) to hunt for interologs in organism p (Fig. 7). The main message indicated by the results for this latter experiment (#4) stays the same as in our original procedure (Methods): Intra species comparisons are more accurate than inter-species comparisons. Due to more samples in the dataset for organism o (Fig. 7) and thus higher counts, the errors slightly decreased.
