# Supplementary material for: Protein–Protein Interactions More Conserved within Species than across Species
Source: PLoS Comput Biol. 2006 Jul 21;2(7):e79. doi: 10.1371/journal.pcbi.0020079 (PMC1513270; doi:10.1371/journal.pcbi.0020079)
Supplement: Supplementary file 3 [file pcbi.0020079.st001.doc]

Table S1: Large-scale protein-protein interaction data sets from IntAct [1] ^∆^

| Dataset | Organism | Method | Number of pair interactions |
| --- | --- | --- | --- |
| Ito (2001) [2] | *Saccharomyces cerevisiae* | Y2H | 4524 |
| Uetz (2000) [3] | *Saccharomyces cerevisiae* | Y2H | 1495 |
| Ho (2002) [4] | *Saccharomyces cerevisiae* | MS | 29098 |
| Gavin (2002) [5] | *Saccharomyces cerevisiae* | TAP | 19254 |
|  |  |  |  |
| Giot (2003) [6] | *Drosophila melanogaster* | Y2H | 20667 |
| Stanyon (2004) [7] | *Drosophila melanogaster* | Y2H | 1615 |
| Formstecher (2005) [8] | *Drosophila melanogaster* | Y2H | 1674 |
|  |  |  |  |
| Li (2004) [9] | *Caenorhabditis elegans* | Y2H | 4037 |
|  |  |  |  |
| Bouwmeester (2004) [10] | *Homo sapiens* | TAP + others | 1670 |

∆	Numbers as taken unfiltered directly from IntAct [1].

# References for 'Supporting online material'

1. Hermjakob H, Montecchi-Palazzi L, Lewington C, Mudali S, Kerrien S, et al. (2004) IntAct - an open source molecular interaction database. Nucleic Acids Res 32: D452-455.

2. Ito T, Chiba T, Ozawa R, Yoshida M, Hattori M, et al. (2001) A comprehensive two-hybrid analysis to explore the yeast protein interactome. Proc Natl Acad Sci U S A 98: 4569-4574.

3. Uetz P, Giot L, Cagney G, Mansfield TA, Judson RS, et al. (2000) A comprehensive analysis of protein-protein interactions in Saccharomyces cerevisiae. Nature 403: 623-627.

4. Ho Y, Gruhler A, Heilbut A, Bader GD, Moore L, et al. (2002) Systematic identification of protein complexes in Saccharomyces cerevisiae by mass spectrometry. Nature 415: 180-183.

5. Gavin AC, Bosche M, Krause R, Grandi P, Marzioch M, et al. (2002) Functional organization of the yeast proteome by systematic analysis of protein complexes. Nature 415: 141-147.

6. Giot L, Bader JS, Brouwer C, Chaudhuri A, Kuang B, et al. (2003) A protein interaction map of Drosophila melanogaster. Science 302: 1727-1736.

7. Stanyon CA, Liu G, Mangiola BA, Patel N, Giot L, et al. (2004) A Drosophila protein-interaction map centered on cell-cycle regulators. Genome Biol 5: R96.

8. Formstecher E, Aresta S, Collura V, Hamburger A, Meil A, et al. (2005) Protein interaction mapping: a Drosophila case study. Genome Res 15: 376-384.

9. Li S, Armstrong CM, Bertin N, Ge H, Milstein S, et al. (2004) A map of the interactome network of the metazoan C. elegans. Science 303: 540-543.

10. Bouwmeester T, Bauch A, Ruffner H, Angrand PO, Bergamini G, et al. (2004) A physical and functional map of the human TNF-alpha/NF-kappa B signal transduction pathway. Nat Cell Biol 6: 97-105.
